# Comprehensive Analysis of Uric Acid and Myasthenia Gravis: IGF1R as a Protective Factor and Potential Therapeutic Target

**DOI:** 10.1111/cns.70361

**Published:** 2025-03-28

**Authors:** Yu Shen, Lijun Pang, Han Wang, Qili Han, Wang Wan, Si Luo, Ziwei Song, Yaofeng Fang, Hao Chen, Yusen Qiu, Dandan Tan, Meihong Zhou, Daojun Hong

**Affiliations:** ^1^ Department of Neurology, The First Affiliated Hospital, Jiangxi Medical College Nanchang University Nanchang China; ^2^ College of Pharmacy Guangxi Medical University Nanning China; ^3^ Rare Disease Center, The First Affiliated Hospital, Jiangxi Medical College Nanchang University Nanchang China; ^4^ Key Laboratory of Rare Neurological Diseases of Jiangxi Provincial Health Commission Nanchang China

**Keywords:** bioinformatics analysis, mendelian randomization, molecular docking, myasthenia gravis, type 1 insulin‐like growth factor receptor, uric acid

## Abstract

**Background:**

Previous studies have suggested that oxidative stress can significantly damage acetylcholine receptors (AChRs), which are implicated in the pathogenesis of myasthenia gravis (MG). Uric acid (UA), a scavenger of peroxynitrite and a natural antioxidant, plays a crucial role in eliminating free radicals in the bloodstream. However, the relationship between UA and MG, as well as the underlying mechanisms, remains insufficiently explored.

**Methods:**

A meta‐analysis was conducted to evaluate the clinical correlation between UA and MG. Subsequently, Mendelian randomization (MR) and bioinformatics analyses were employed to identify the key protein IGF1R. Public datasets, such as TCGA and GEO, along with patient data from our clinical center, were used for a comprehensive analysis of the relationship between IGF1R and UA in MG patients. Additionally, virtual screening and molecular docking were performed to identify small molecules that target IGF1R as potential therapeutic agents for MG.

**Results:**

The meta‐analysis revealed a significant association between low UA levels and MG (OR −48.46 [95% CI −63.26, −33.65], *p* < 0.00001). The two‐sample MR analysis indicated a genetic relationship between UA and MG (*p* = 0.024; *p* = 0.036). The FUMA analysis and enrichment analysis identified IGF1R as a key protein likely involved in this relationship. Using the thymoma dataset from the TCGA database, we analyzed IGF1R expression in the MG and non‐MG groups and found that IGF1R expression was lower in MG patients and was associated with a poor prognosis (*p* < 0.05). Single‐cell RNA‐seq data from the GEO database further supported the association between low IGF1R expression and MG, as well as the occurrence of crisis (*p* < 0.05). Additionally, data from MG patients treated at our center showed that IGF1R expression correlated with UA levels and that higher IGF1R expression was associated with milder clinical phenotypes (ocular phenotypes). Through a virtual screen and molecular docking of small molecules in the DrugBank database, we identified several potential small‐molecule drugs that may target IGF1R to treat MG.

**Conclusions:**

Our study revealed an association between low UA levels and MG and subsequently showed that low IGF1R expression is associated with the onset, severity, and poor prognosis of MG. We also explored the molecular mechanisms underlying the protective role of IGF1R in MG and identified potential drugs for treating MG.

## Graphical abstract

This study used meta‐analysis to establish a correlation between low UA levels and MG. Furthermore, MR and bioinformatics analyses identified IGF1R as a key protein in this relationship. The correlation between IGF1R and MG was further validated using the TCGA dataset, GEO single‐cell RNA sequencing datasets, and clinical data from MG patients, which indicated that low IGF1R expression is associated with MG. Finally, several small‐molecule drugs targeting IGF1R were identified, offering potential therapeutic options for MG.

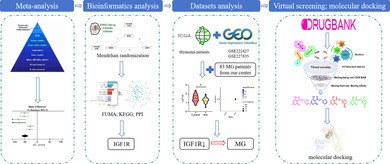

## 1 Introduction

Myasthenia Gravis (MG) is a common neuromuscular disorder caused by autoantibodies targeting postsynaptic acetylcholine receptors (AChRs), resulting in impaired neuromuscular junction (NMJ) transmission [1]. In addition to AChR antibodies, other antibody subtypes, such as muscle‐specific kinase (MuSK) antibodies, contribute to its pathogenesis [2]. Emerging evidence suggests that oxidative stress plays a significant role in the immune pathogenesis of neuromuscular diseases [3, 4].

Reactive oxygen species (ROS) are highly reactive oxygen‐containing molecules, including free radicals (e.g., superoxide and hydroxyl radicals) and nonradicals (e.g., hydrogen peroxide) [5]. Despite their structural differences, these ROS share a common mechanism of cellular damage by targeting proteins, DNA, and lipids [5]. Antioxidant defense systems, both enzymatic and nonenzymatic, help clear or reduce ROS levels, maintaining cellular redox homeostasis [6]. Among these antioxidants, uric acid (UA) functions as a scavenger of peroxynitrite and a natural antioxidant, accounting for 60% of the free radical‐scavenging activity in human blood [7, 8, 9]. Oxidative radicals have been implicated in neuromuscular damage across various disorders, with sustained free radical activity shown to damage AChRs significantly [10]. Previous studies have demonstrated the therapeutic effects of UA on experimental autoimmune encephalomyelitis and its potential benefits in patients with multiple sclerosis (MS) [7, 11]. However, the relationship between UA and MG, as well as the underlying mechanisms, remains underexplored.

Several small‐scale clinical studies have noted reduced UA levels in MG patients, with lower UA levels associated with a more severe MGFA (MG Foundation of America) classification [12]. Comprehensive evaluations of this relationship and mechanistic insights are lacking. This study aims to bridge these gaps by employing a meta‐analysis to assess the clinical relationship between UA and MG, alongside Mendelian randomization (MR) to elucidate genetic associations and identify key mediating genes (type 1 insulin‐like growth factor receptor; IGF1R). Using datasets from the Gene Expression Omnibus (GEO) and The Cancer Genome Atlas (TCGA), we validated the relationship between IGF1R and MG. Additionally, single‐center data were utilized to comprehensively evaluate the clinical associations of IGF1R with UA and MG. Finally, virtual screening was employed to identify small‐molecule compounds targeting IGF1R as potential therapeutic candidates for MG. Through this study, we aimed to elucidate the molecular mechanisms underlying the protective role of IGF1R in MG and identify potential therapeutic drugs.

## 2 Methods

### 2.1 Meta‐Analysis

A literature search was conducted independently by two neurologists. A systematic search of the PubMed, EMBASE, Cochrane Library, and Web of Science databases was performed, covering publications from database inception to June 2024. The search keywords used were “myasthenia gravis” and “uric acid.” Reviews, conference abstracts, case reports, and animal studies were excluded from this analysis. Duplicate data or publications were also excluded, along with studies lacking sufficient information. Data extraction was performed independently by two authors, who collected the following details: (1) Basic information: author, article title, publication date, and study population. (2) UA levels. (3) Sample size: the number of MG patients and controls. Any discrepancies between the two reviewers were resolved by a third expert. The detailed criteria for the literature evaluation and modeling analysis methods are outlined in our previous publication [13]. Meta‐analyses were conducted using Review Manager (RevMan) version 5.3 software.

### 2.2 MR, FUMA Analysis, and PPI Network Construction

The two‐sample MR approach was designed to test the causal relationship between UA as the exposure factor and MG as the outcome factor. Independent single nucleotide polymorphisms (SNPs) from genome‐wide association studies (GWASs) served as instrumental variables (IVs). In accordance with the MR analysis requirements, exposure and outcome datasets were obtained from the IEU Open GWAS project database (https://gwas.mrcieu.ac.uk). The genetic phenotype “UA” was searched in the IEU Open GWAS database, yielding two UA datasets: ebi‐a‐GCST90018977 and ebi‐a‐GCST90018757 from European and Asian populations, respectively. Additionally, an MG dataset (finn‐b‐G6_MYASTHENIA) was included. The detailed methods used for SNPs selection, analysis, and heterogeneity evaluation can be found in our previous studies [14, 15]. The identified SNPs were subsequently matched to corresponding genes using FUMA analysis. Subsequently, KEGG pathway and protein‐protein interaction (PPI) network analyses were performed to identify potential key genes and pathways. The intersection of key genes from European and Asian populations was used to determine the final candidate genes for further investigation.

### 2.3 TCGA and GEO Public Dataset Analysis

Relevant data were sourced from the TCGA database (https://portal.gdc.cancer.gov/), which includes RNA sequencing data and clinical information from 93 thymoma patients (32 diagnosed with MG), to validate the expression of the key genes identified to be involved in MG. The data were analyzed by calculating the fragments per kilobase of transcript per million fragments to profile mRNA expression. Additionally, single‐cell RNA sequencing data were obtained from the National Center for Biotechnology Information (NCBI) GEO databases (https://www.ncbi.nlm.nih.gov/geo/, ID: GSE222427, GSE227835). Corresponding datasets were sought to explore the potential differences in IGF1R expression levels before and after MG treatment. The GSE222427 dataset includes data from 3 MG patients, with single‐cell sequencing performed before and after MG crisis treatment [16]. We searched for eligible datasets with the largest sample sizes to explore the differences in IGF1R levels between the MG and control groups. The GSE227835 dataset includes single‐cell RNA sequencing data from 10 MG patients and 10 negative controls [17]. A comprehensive analysis of the IGF1R mRNA expression levels in MG patients and changes before and after treatment was performed using three public datasets. TCGA data were analyzed, and quality control, clustering, and gene expression analyses of the single‐cell data were also performed using R software (version 4.2.1).

### 2.4 Expression of IGF1R and Its Relationship With UA Levels and the Clinical Manifestations of MG

This study prospectively collected data from MG patients at the Department of Neurology, the First Affiliated Hospital of Nanchang University, from January 2023 to January 2024. The study was approved by the Ethics Committee of the First Affiliated Hospital of Nanchang University (AG‐SG‐03‐2.1‐IIT). The data collected included routine clinical information, as well as the clinical manifestations of MG, such as the age of onset, disease duration, clinical outcome, and whether a crisis had occurred. Data on the presence of thymoma, blood UA levels, and MGFA scores were also collected. The exclusion criteria included patients with hyperuricemia (serum UA levels > 420 μmol/L), and blood samples were obtained with patient consent. The blood samples were used to detect IGF1R levels using an enzyme‐linked immunosorbent assay (ELISA). Univariate categorical data were analyzed using the chi‐square test or Fisher's exact test. For continuous variables, differences in continuous variables between two groups were detected using the Shapiro–Wilk test to assess whether the data obeyed a normal distribution. Normally distributed data were analyzed using independent *t*‐tests, while non‐normally distributed data were analyzed using the Mann–Whitney *U* test. A *p*‐value of < 0.05 was considered statistically significant. All statistical analyses were performed using IBM SPSS Statistics 25.0 (SPSS, Chicago, IL, USA).

### 2.5 Small Molecule Drugs for the Possible Treatment of MG and Molecular Docking

In this study, the structure of IGF1R (protein ID: P08069) was obtained from the AlphaFoldDB database. The structure was first processed by removing water molecules, ligands, and irrelevant ions to meet the docking requirements of AutoDock Vina. Hydrogen atoms were added, and charges were computed using AutoDock Tools. This study utilized the predicted active pocket information of IGF1R from the Pocasa tool (https://g6altair.sci.hokudai.ac.jp/g6/service/pocasa/) for docking to enhance the docking accuracy. The ligands were sourced from the DrugBank database, which includes small molecules both used in clinical practice and under experimental investigation. The small molecule structures were converted into 3D coordinates using Open Babel, followed by molecular optimization with Avogadro. Hydrogen atoms were added, and charge calculations were performed to ensure that the resulting pdbqt files met AutoDock Vina requirements. The docking parameters were set as follows: receptor protein structure center coordinates—center_x = −13.5, center_y = 13.0, and center_z = −32.5 (determined based on the predicted active pocket coordinates from Pocasa); grid size—size_x = 29.0, size_y = 22.0, size_z = 29.0 (chosen based on the size of the protein and the distribution of the active pocket); docking precision—exhaustiveness = 4 (a compromise between the calculation precision and time; higher values improve accuracy); the number of docking modes—num_modes = 1 (selecting the most likely binding mode for each ligand‐receptor pair); energy range—energy_range = 2 kcal/mol (allowed energy deviation for the binding mode); and random seed—seed = 20,241,120 (ensuring randomness in the docking experiment). These settings ensured that the docking calculations focused on the most probable regions required for IGF1R binding, thus improving the efficiency and accuracy of the screening process. The docking results for all the ligands were ranked according to the computed binding affinity, with lower binding energies indicating stronger ligand‐receptor affinity. The screening criterion for potential candidate drugs was set to retain ligands with a binding energy lower than −10 kcal/mol. The spatial conformation of the complex was analyzed using PyMOL. By examining the binding sites, we assessed their stability and activity. Special attention was given to whether the ligand successfully entered the receptor's binding pocket and formed key hydrogen bonds and hydrophobic interactions, ensuring stable binding.

## 3 Results

### 3.1 Meta‐Analysis

**FIGURE 1:**
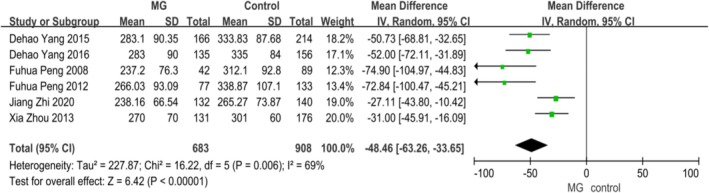
Forest plot showing the relationship between myasthenia gravis and uric acid levels.

Through a systematic search of the PubMed, Embase, Web of Science, and Cochrane Library databases, a total of 7 articles were identified. We screened the titles and abstracts, and 1 article was excluded because of irrelevance. After a thorough review of the abstracts and full texts, 6 studies were included in the analysis, comprising 683 MG patients and 908 controls. The characteristics of the studies included in the meta‐analysis are shown in Table 1 [10, 12, 18, 19, 20]. Due to an *I* ^2^ of 69%, a random‐effects model was used for the meta‐analysis. The results indicated that the serum UA level in MG patients was significantly lower than that in the controls (OR −48.46 [95% CI −63.26, −33.65], *p* < 0.00001) (Figure 1).

**TABLE 1:** Characteristics of included studies in meta‐analysis.

References	MG (*N*)	Mean ± SD	Control (*N*)	Mean ± SD
Dehao Yang (2016)	135	283 ± 90	156	335 ± 84
Fuhua Peng (2008)	42	237.2 ± 76.30	89	312.1 ± 92.80
Fuhua Peng (2012)	77	266.03 ± 93.09	133	338.87 ± 107.10
Jiang Zhi (2020)	132	238.16 ± 66.54	140	265.27 ± 73.87
Dehao Yang (2015)	166	283.10 ± 90.35	214	333.83 ± 87.68
Xia Zhou (2013)	131	270 ± 70	176	301 ± 60

Abbreviations: MG, Myasthenia Gravis; SD, standard deviation.

### 3.2 Identification of Key Genes via MR and Bioinformatic Analyses

**FIGURE 2:**
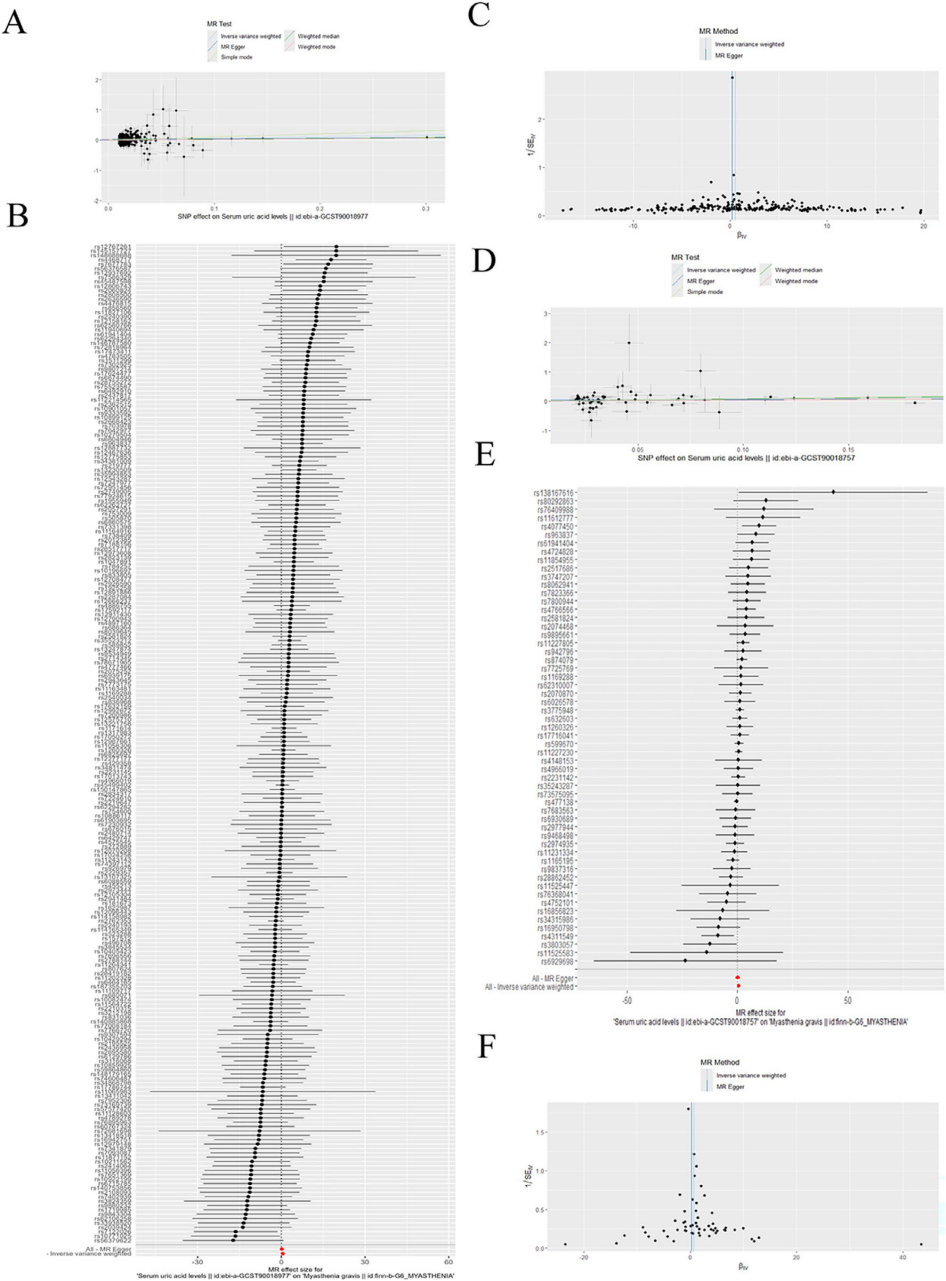
Two‐sample Mendelian Randomization analysis of myasthenia gravis and uric acid. (A, D) Scatter plots; (B, E) Forest map; (C, F) Funnel plot.

**FIGURE 3:**
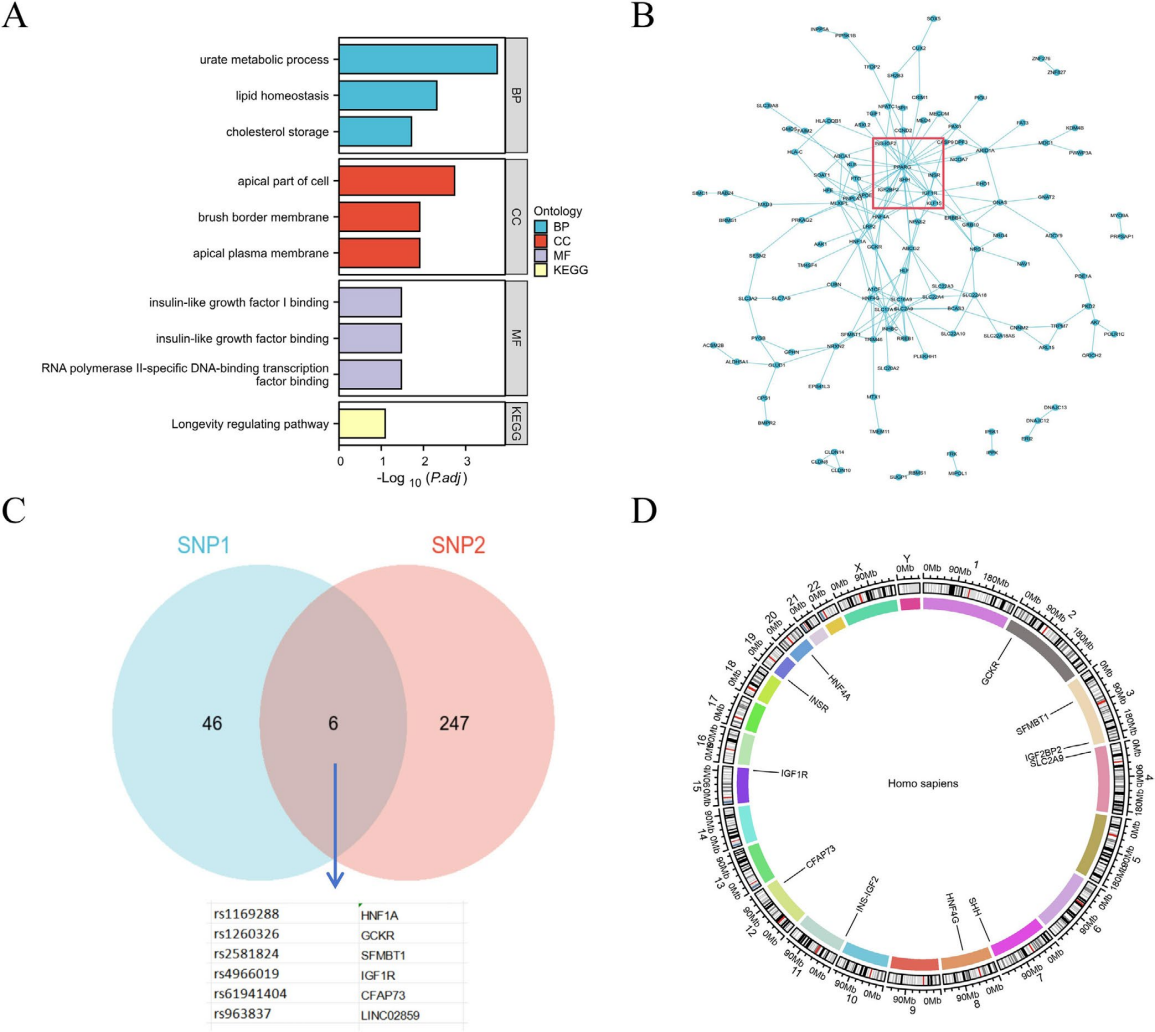
Bioinformatics analysis to identify key genes associated with uric acid and myasthenia Gravis. (A) KEGG/GO analysis showing biological processes, cellular components, and molecular functions, with molecular functions particularly focusing on IGF1. (B) PPI network analysis, where IGF and related genes are positioned at the center of the network (red box). (C) Venn diagram showing the intersection of Mendelian Randomization analysis results from the Asian dataset (SNP1) and the European dataset (SNP2), with six shared genes from the uric acid dataset. (D) Chromosome location map of the genes, showing that the IGF1R gene is located on chromosome 15.

We investigated the genetic relationship between UA and MG by first selecting UA datasets from open GWAS databases; ebi‐a‐GCST90018977 (European population) and ebi‐a‐GCST90018757 (Asian population) were included. The MG dataset was obtained from the Finnish database, specifically finn‐b‐G6_MYASTHENIA, for use in this study. For the ebi‐a‐GCST90018977 dataset, the inverse‐variance weighted (IVW) analysis showed a genetic causal relationship between UA and MG (OR = 1.723; 95% CI 1.072–2.772; *p* = 0.024) (Figure 2A–C). Similarly, the ebi‐a‐GCST90018757 dataset yielded consistent results, suggesting a causal relationship (OR = 1.944; 95% CI 1.045–3.617; *p* = 0.036) (Figure 2D–F). Additionally, heterogeneity and pleiotropy were assessed in the MR studies. For the ebi‐a‐GCST90018977 dataset, no significant heterogeneity (*P*_MR‐Egger_ = 0.812, *P*_MR‐IVW_ = 0.797) or horizontal pleiotropy (*P*_MR‐Egger_ = 0.148, *P*_MR‐PRESSO_ = 0.823) was observed. For the ebi‐a‐GCST90018757 dataset, no significant heterogeneity (*P*_MR‐Egger_ = 0.188, *P*_MR‐IVW_ = 0.210) or horizontal pleiotropy (*P*_MR‐Egger_ = 0.464, *P*_MR‐PRESSO_ = 0.478) was observed. A total of 253 SNPs were identified in the dataset from the European population, whereas 52 SNPs were identified in the dataset from the Asian population. These SNPs were mapped to corresponding genes using independent GWAS SNPs and linkage disequilibrium (LD) filtering, resulting in the identification of 234 genes. KEGG/GO enrichment and PPI network analyses were then performed. The biological process (BP) results highlighted UA metabolism as the primary process, whereas insulin‐like growth factor 1 binding emerged as the key molecular function (Figure 3A). The PPI network analysis further pinpointed central genes, such as *PPARG*, *IGF1R*, and *IGF2BP2* (Figure 3B). The intersection of genes from Asian and European populations revealed *HNF1A*, *GCKR*, *SFMBT1*, *IGF1R*, *CFAP73*, and *LINC02859* as shared genes (Figure 3C). According to the results of the KEGG/GO enrichment analyses, insulin‐like growth factor 1 binding is a key molecular function, and in the PPI network analysis, *IGF1R* was selected as the key gene for further investigation in this study (Figure 3D).

### 3.3 IGF1R Expression Levels in MG Patients From Multiple Databases

**FIGURE 4:**
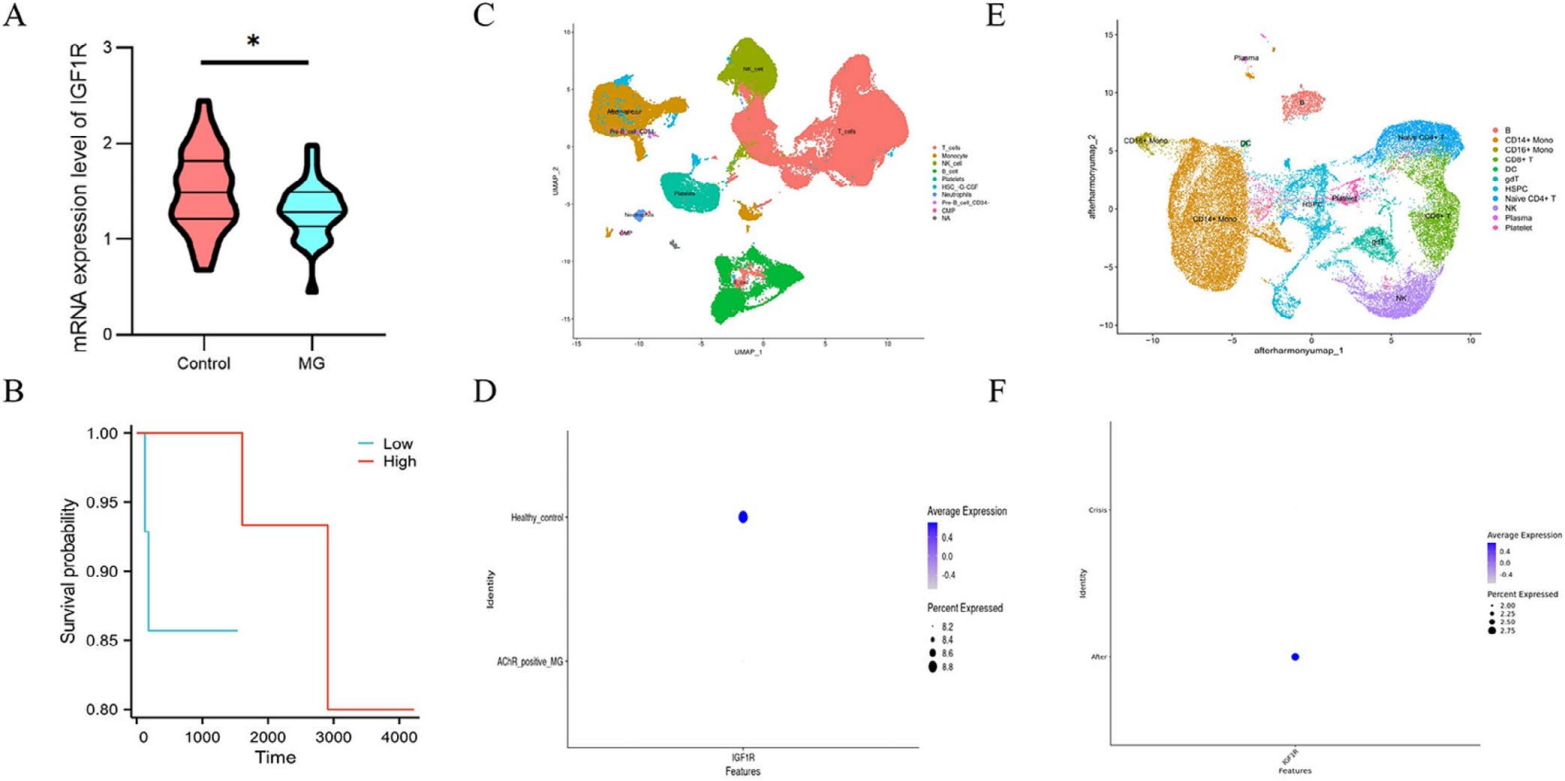
Validation of the relationship between IGF1R and myasthenia gravis using public datasets. (A, B) Comparison of mRNA expression levels of IGF1R between the myasthenia gravis and non‐myasthenia gravis groups in the TGCA thymoma dataset (A). The expression level of IGF1R was categorized into two groups based on the median, and higher expression was associated with better clinical prognosis (B). (C, D) UMAP plot of single‐cell sequencing cell clustering in the GSE227835 dataset from GEO database (C). IGF1R expression was lower in myasthenia gravis patients (D). (E, F) UMAP plot of single‐cell sequencing cell clustering in the GSE222427 dataset from GEO database (E). IGF1R expression was lower in myasthenia gravis crisis patients (F). *Note*: *p < 0.05.

We analyzed the thymoma dataset from the TCGA database and divided it into two groups to evaluate IGF1R expression in MG patients: patients diagnosed with MG (*n* = 32) and non‐MG patients (*n* = 61). The analysis revealed a significant reduction in IGF1R mRNA expression in MG patients compared with non‐MG patients (*p* < 0.05) (Figure 4A). Next, a prognostic analysis was performed. IGF1R expression levels were stratified by median values into high‐expression and low‐expression groups. The Cox regression analysis revealed that higher IGF1R expression was correlated with improved survival outcomes (Figure 4B). Further validation was conducted using single‐cell sequencing data from the GEO database. In the GSE227835 dataset, which includes 10 MG patients (AChR antibody‐positive) and 10 control individuals, a comparative analysis confirmed significantly reduced IGF1R expression in MG patients (*p* < 0.05) (Figure 4C,D). Additionally, single‐cell sequencing data from patients who underwent MG crisis treatment revealed an increase in IGF1R expression after treatment across multiple cell populations. The total IGF1R expression levels were also significantly increased post‐treatment (*p* < 0.05) (Figure 4E,F). These findings highlight the potential roles of IGF1R in MG pathophysiology and prognosis, providing insights into therapeutic targets and disease mechanisms.

### 3.4 High IGF1R Expression Is Associated With a Milder Clinical Phenotype in MG Patients

**FIGURE 5:**
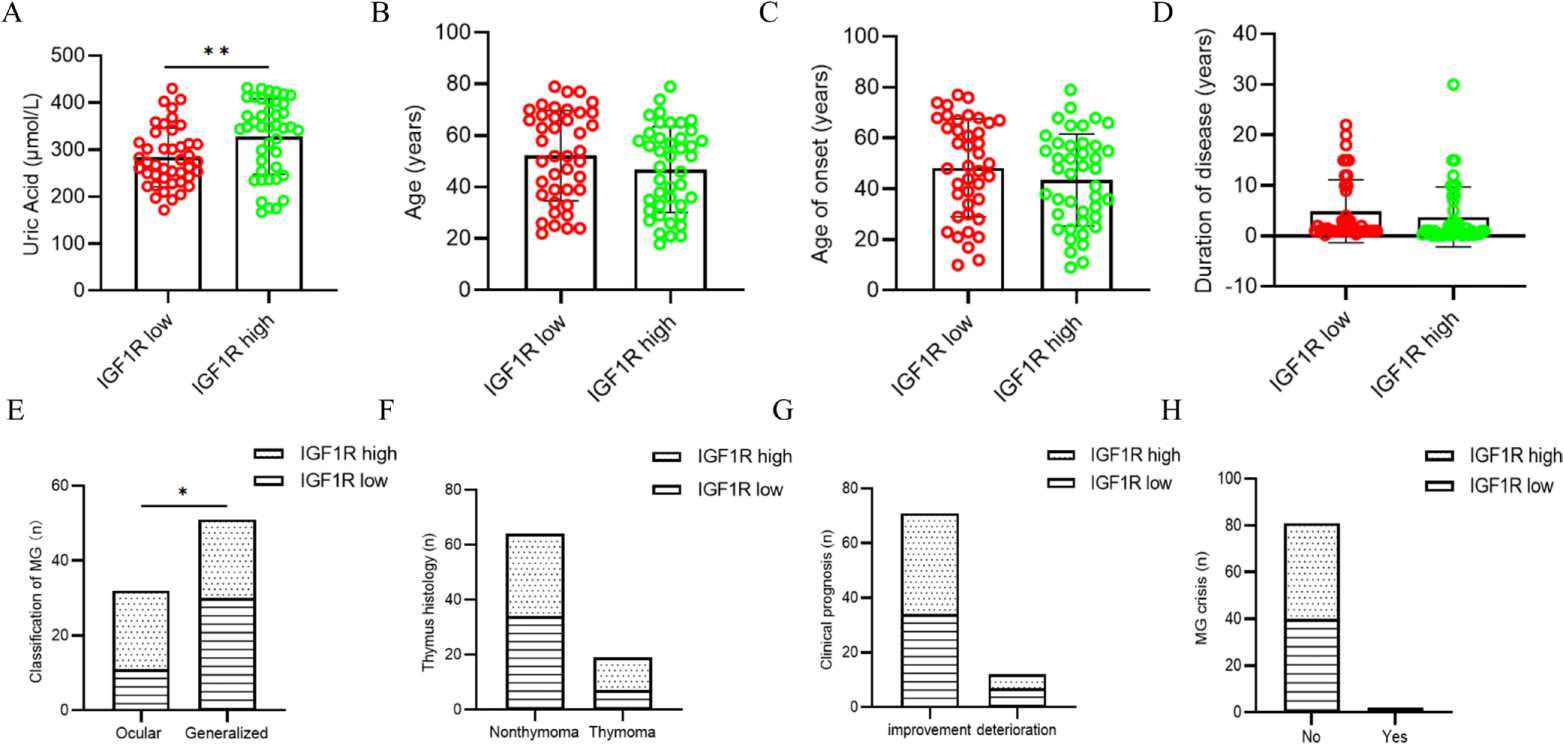
Comparison of clinical manifestations in myasthenia gravis patients with high and low expression of IGF1R in a single clinical center (unpaired *t*‐test/chi‐square test). *Note*: *p < 0.05, **p < 0.01.

A total of 83 MG patients who consented to provide whole blood samples were prospectively recruited at our medical center. Serum UA and IGF1R levels were measured in all participants. The cohort was divided into two groups according to the median expression level of IGF1R. The low‐IGF1R group (*n* = 41) included 12 males (29.3%), with a mean age of 52.2 ± 17.56 years. The high‐IGF1R group (*n* = 42): included 28 males (66.7%) with a mean age of 46.86 ± 16.67 years (Table 2). A significant difference in the sex distribution was observed between the two groups, with the high IGF1R group having a greater proportion of males (*p* < 0.05). Compared with those in the low‐IGF1R group, serum UA levels were significantly increased in the high‐IGF1R group (*p* < 0.05) (Figure 5A). Clinical characteristics and outcomes were also compared between the groups. The results revealed a significantly greater proportion of ocular MG cases in the high IGF1R group, whereas the MGFA score indicated a greater proportion of mild cases in this group (*p* < 0.05) (Figure 5F). However, no significant differences were observed between the two groups in the age of onset, disease duration, presence of an MG crisis, treatment outcomes, or incidence of thymoma (*p* > 0.05) (Figure 5B–D,F–H). These findings suggest a potential relationship between IGF1R expression, UA levels, and the clinical presentation of MG. IGF1R is also thought to be associated with a milder clinical phenotype in MG patients.

**TABLE 2:** Comparison of clinical features of myasthenia gravis patients with IGF1R low and IGF1R high.

Characteristics	IGF1R low (*n* = 41)	IGF1R high (*n* = 42)	*p*
Sex, *n* (%)			**0.0009**
Male	12 (29.3%)	28 (66.7%)	
Female	29 (70.7%)	14 (33.3%)	
Age (years)	52.2 ± 17.56	46.86 ± 16.67	0.1593
Classification of MG, *n* (%)			**0.0424**
Ocular	11 (26.8%)	21 (50%)	
Generalized	30 (73.1%)	21 (50%)	
Thymus histology, *n* (%)			0.2969
Nonthymoma	34 (82.9%)	30 (71.4%)	
Thymoma	7 (17.1%)	12 (28.6%)	
Clinical prognosis, *n* (%)			0.5477
Improvement	34 (82.9%)	37 (88.1%)	
Deterioration	7 (17.1%)	5 (11.9%)	
MG crisis, *n* (%)			> 0.9999
No	40 (97.6%)	41 (97.6%)	
Yes	1 (2.4%)	1 (2.4%)	
Age of onset (years)	48.27 ± 19.32	43.48 ± 18.14	0.2473
Duration of disease (years)	4.698 ± 6.367	3.598 ± 6.024	0.4211
MGFA			
I	10 (24.4%)	24 (57.1%)	**0.0390**
II	19 (46.3%)	11 (26.2%)	
III	4 (9.8%)	1 (2.4%)	
IV	2 (4.9%)	2 (4.8%)	
V	6 (14.6%)	4 (9.5%)	

*Note:* Bold values indicate statistical significance at *p* < 0.05.

Abbreviations: MG, Myasthenia Gravis; MGFA, Myasthenia Gravis Foundation of America. *Note:* Bold values indicate statistical significance at *p* < 0.05.

### 3.5 Small Molecule Drugs and Molecular Docking

**FIGURE 6:**
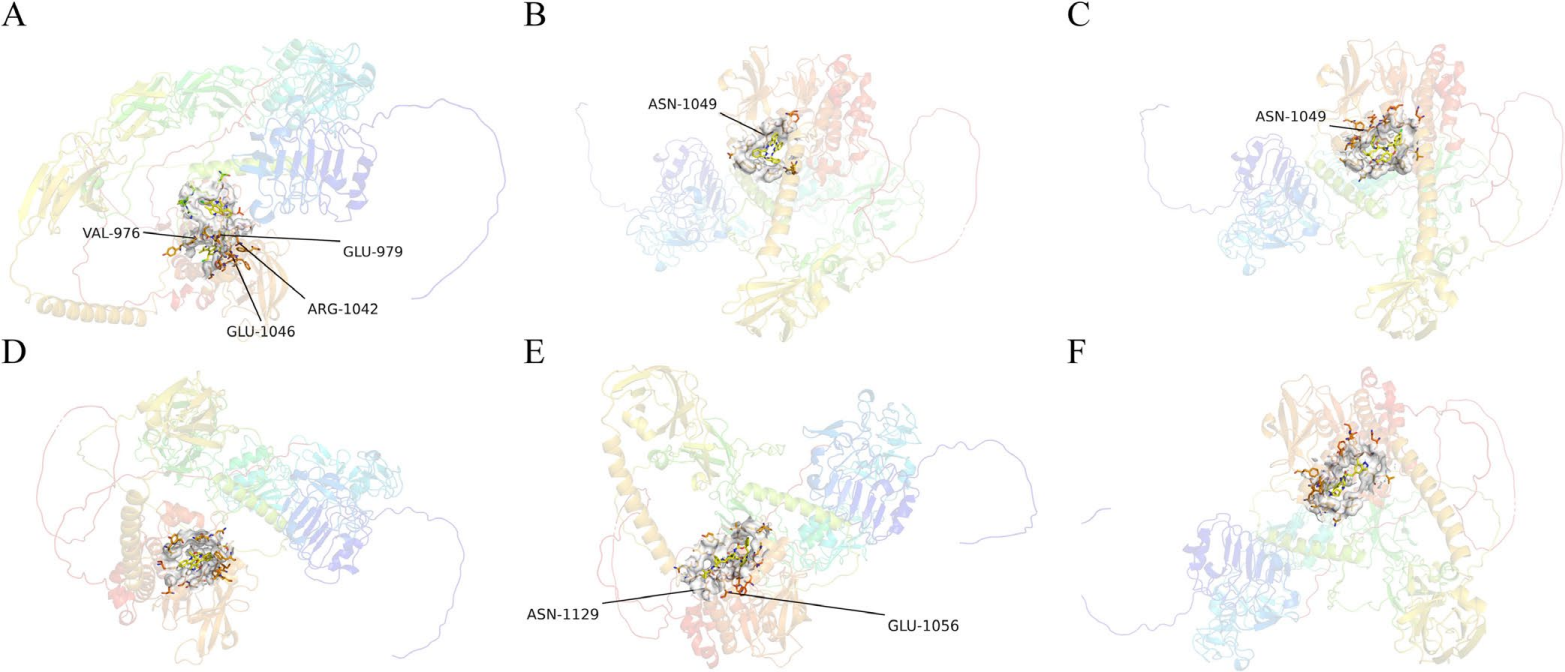
Molecular docking of IGF1R with small molecules identified through virtual screening. (A) Bizelesin; (B) Phthalocyanine; (C) Murizatoclax; (D) Midostaurin; (E) MK‐6325; (F) Zavegepant.

Through a molecular docking screen of all small molecules from the DrugBank database, the top 10 ligands with the strongest binding affinity to IGF1R were successfully identified (Table 3). Bizelesin's binding mode with IGF1R primarily relies on hydrogen bonds and hydrophobic interactions. This ligand forms multiple hydrogen bonds with key amino acid residues (VAL‐976, GLU‐979, ARG‐1042, and GLU‐1046) in the active site of IGF1R and strongly interacts with the protein surface in the hydrophobic region. This drug may regulate IGF1R activity by stabilizing binding (Figure 6A). The planar structure of Phthalocyanine allows it to fit tightly into the hydrophobic pocket of IGF1R. This ligand primarily interacts with the hydrophobic region of IGF1R through hydrophobic interactions and specifically binds to the key amino acid residue ASN‐1049. This mechanism may play a role in regulating IGF1R function (Figure 6B). Murizatoclax also forms a stable complex with IGF1R via both hydrophobic interactions and hydrogen bonds, particularly by stabilizing the interaction through hydrogen bonding with the IGF1R residue ASN‐1049. Its binding energy is similar to that of Phthalocyanine, indicating a similar binding affinity, and it may regulate IGF1R through a similar mechanism (Figure 6C). Midostaurin binds to the active pocket of IGF1R primarily through hydrophobic interactions with its hydrophobic region without significantly forming hydrogen bonds. It shows a medium binding affinity. As a multi‐target kinase inhibitor already applied in the treatment of certain leukemias, the antitumor effects of Midostaurin could be enhanced by interacting with IGF1R (Figure 6D). MK‐6325 binds to the hydrophobic pocket of IGF1R and interacts with residues ASN‐1129 and GLU‐1056 via hydrogen bonds. With a binding energy of −10.5 kcal/mol, this ligand has significant binding affinity and may affect IGF1R function and regulate its signaling pathways (Figure 6E). Zavegepant primarily interacts with the hydrophobic region of IGF1R and stably binds to its active site. With a binding energy of −10.5 kcal/mol, Zavegepant may play a role in the regulation of IGF1R, particularly in the modulation of the IGF1R signaling pathway (Figure 6F). All these small molecules may have the potential to exert pharmacological effects on the treatment of MG by modulating IGF1R.

**TABLE 3:** Ranking of Top 10 binding energies of IGF1R and small molecule drugs.

Ranking	Ligand name	Energy (kcal/mol)
1	Bizelesin	−11.3
2	Phthalocyanine	−10.7
3	Murizatoclax	−10.7
4	Midostaurin	−10.5
5	MK‐6325	−10.5
6	Zavegepant	−10.5
7	Tapotoclax	−10.4
8	BMS‐986142	−10.3
9	1370466‐81‐1	−10.3
10	SD146	−10.2

## 4 Discussion

**FIGURE 7:**
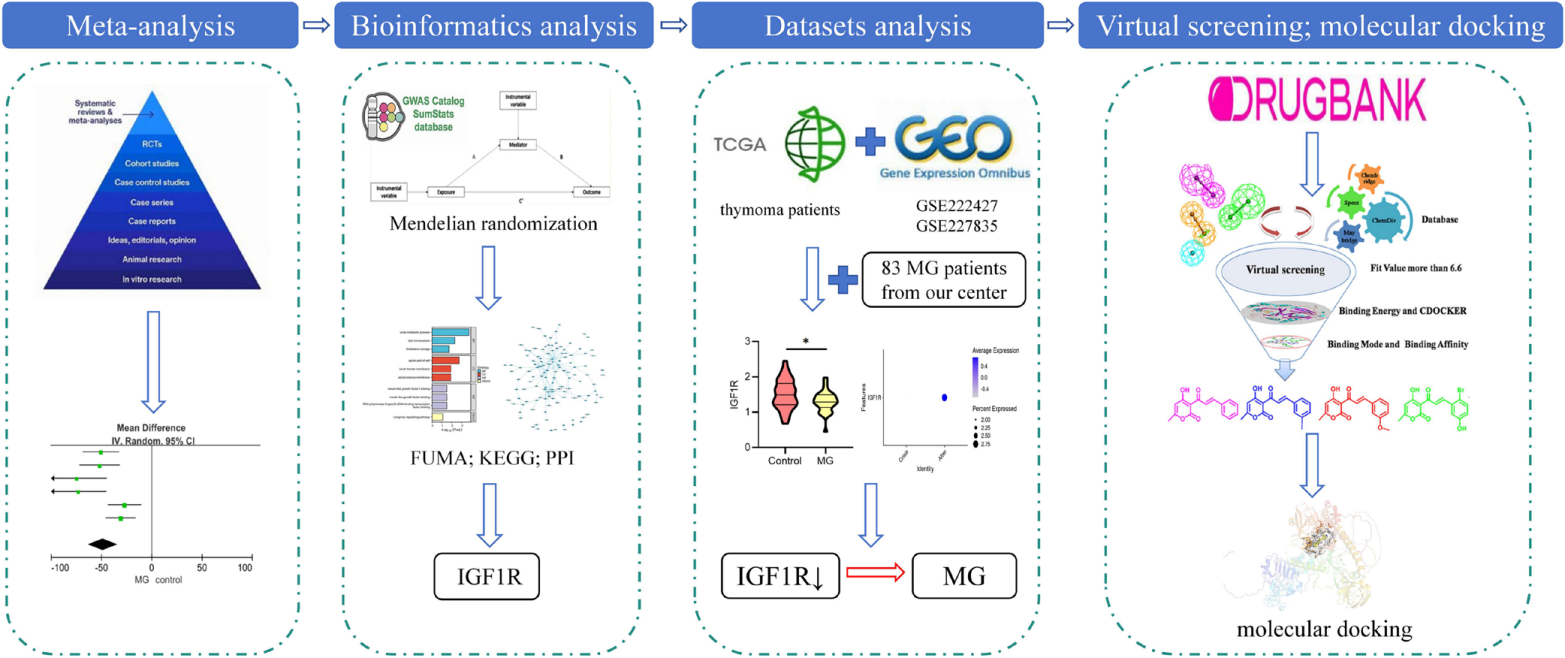
The figure summarizes the full process of the study, beginning with the meta‐analysis, followed by bioinformatics analysis and database validation, and concluding with the drug screening and molecular docking.

This study revealed a correlation between low uric acid levels and MG. In addition, the bioinformatic analysis indicated that IGF1R is a key protein involved in this relationship. The use of single‐cell RNA sequencing datasets and clinical data from MG patients further suggested that low expression of IGF1R is associated with MG, suggesting that IGF1R may play a protective role in this disease. Finally, several small‐molecule drugs targeting IGF1R have been identified, providing potential therapeutic options for MG (Figure 7).

MG is an acquired autoimmune disease caused by impaired NMJ transmission, typically due to autoantibodies against skeletal muscle AChRs on the postsynaptic membrane [21]. In more than 80% of known cases, MG is caused by autoantibodies against AChRs [22, 23]. In patients who are negative for AChR antibodies, some may be positive for antibodies against MuSK or LRP4 [2, 24]. In addition to the involvement of various autoantibodies, MG may also be associated with the loss of immune tolerance caused by thymic damage [25]. Moreover, complement proteins and cytokines play roles in the autoimmune process [26]. Although the primary pathological site of MG is the NMJ, studies suggest that long‐term immune attack by multiple autoantibodies can cause muscle cell damage, even leading to muscle fiber degeneration and atrophy [27]. As a result, MG patients often experience subclinical muscle damage, which may impair the ability of the muscle to repair itself. Under normal circumstances, mild muscle injury triggers a protective repair process, but in MG patients, immune interference may affect muscle regeneration [28]. Research has shown that the function of muscle satellite cells in MG patients may be suppressed, hindering muscle repair processes [28]. Therefore, the development of MG involves both muscle damage and a decrease in the regenerative capacity of the muscle.

*IGF1R* is a receptor‐type tyrosine kinase that is widely expressed on the cell membranes of various tissues in the human body and is involved in regulating several crucial physiological processes, including cell survival, proliferation, apoptosis, and migration [29]. In both humans and animal models, IGF‐1 levels decrease with age, which is consistent with our findings in MG patients, where the low IGF1R group had an older average age [30, 31, 32]. IGF1R has been extensively studied in the context of cancer, where increased IGF‐1R activity is believed to promote the proliferation, migration, and invasion of cancer cells and is associated with tumor metastasis, treatment resistance, and reduced survival rates [33]. However, IGF1R also plays a role in muscle repair and regeneration [34]. The ligand of IGF1R, IGF‐1, is critical in muscle healing and maintenance. Preclinical studies have shown that IGF‐1 is associated with muscle mass and strength development. It can reduce muscle degeneration, prevent the excessive toxin‐induced expansion of inflammation, and increase the proliferative capacity of muscle satellite cells (MSCs) [35]. IGF‐1 is also considered a biomarker of health and adaptability; in fact, higher circulating levels of IGF‐1 are positively correlated with health factors related to body composition and cardiovascular strength and negatively correlated with body fat levels [36]. Aerobic fitness and muscle endurance are positively correlated with circulating IGF‐1 levels [36]. Additionally, research has confirmed that IGF‐1 stimulates the development of neuronal synapses and plays an important role in the development of the central and peripheral nervous systems [37, 38]. It has been shown to exert restorative effects on the brain by promoting hippocampal neurogenesis and improving memory accuracy in older adults and patients with neurodegenerative diseases [39]. Furthermore, studies suggest that the nutritional influence of IGF‐1 on sensory and motor neurons, as well as neuronal growth and recovery, becomes progressively stronger than that of growth hormone [37]. These findings collectively suggest that IGF1R can aid in the repair and regeneration of muscle and nerve structures, thus potentially providing a therapeutic strategy for treating MG.

Based on the hypothesis of the MR analysis, IGF1R expression is associated with UA levels, which in turn affects MG. This hypothesis is supported by our clinical data, which show that lower IGF1R levels are associated with lower UA levels in MG patients. Additionally, research has confirmed that IGF1R expression is clearly correlated with UA levels [40]. However, the exact mechanism by which UA influences the MG remains unclear. Increasing evidence suggests that oxidative stress contributes to the pathogenesis of inflammation and tissue damage mediated by autoimmune processes [41]. Researchers have hypothesized that MG patients exhibit increased levels of oxidative stress, which may lead to AChR damage [10]. Numerous studies have shown that serum UA levels are reduced in patients with inflammatory and autoimmune diseases, a finding that was further confirmed in our study, where UA levels were significantly lower in MG patients [42, 43, 44]. UA is considered an effective substance for eliminating oxidative stress. Research on MS has shown that UA could serve as an alternative marker of disease activity and is an antioxidant [45]. Furthermore, UA treatment has been proven to prevent inflammation and may be effective in both animal models and patients with MS [46]. However, supplementation with UA remains a challenging therapeutic strategy because of its dual nature, which introduces uncertainty regarding its potential negative effects.

The primary therapeutic strategies for MG involve reducing antibody production, clearing antibodies, and mitigating the effects of these antibodies [47]. With advancements in drug research, targeted immunotherapy for MG has shown promise in reducing B cell survival, inhibiting complement activation, and lowering serum IgG concentrations [48]. These approaches have been confirmed in clinical trials, and some drugs have already entered clinical practice. This study identified IGF1R as a potential new therapeutic target for MG. The small molecules screened in this study exhibit high binding affinity for IGF1R, and molecular docking further confirmed the interactions between these drugs and IGF1R. Bizelesin, as a calcium modulator, utilizes its pyrrole ring structure to interact with calcium channels, promoting calcium intake and bone formation, making it a potential therapeutic agent for osteoporosis and metabolic diseases. Phthalocyanine is well‐known for its potent antioxidant properties, and its polycyclic aromatic structure allows it to scavenge free radicals, protecting neurons from oxidative stress, making it applicable in the treatment of neurodegenerative diseases. Murizatoclax, a steroid receptor inhibitor, has a biphenyltriene structure that enables it to block the activation of nuclear receptors, thereby reducing the expression of inflammatory factors. Midostaurin functions by inhibiting the breakdown activity of polypeptidases, and its pyrrole‐benzonic acid structure helps it exert therapeutic effects in cancer and autoimmune diseases. MK‐6325 is closely associated with the NF‐κB signaling pathway. Its pyridoxine group enables it to interact with this transcription factor, modulating immune regulatory functions. Zavegepant, a steroid receptor agonist, features a pyran structure that enables it to activate steroid receptors, thereby improving nerve conduction and muscle contraction strength, making it useful in the treatment of neurological disorders such as multiple sclerosis. Therefore, the identified small molecules are potential candidates for further experimental validation.

This study first highlights the relationship between UA and MG and identifies the potential mechanisms linking IGF1R to MG. These findings were further validated using patient samples and clinical data. However, this study has several limitations: (1) The molecular mechanisms through which IGF1R and UA influence MG have not been fully elucidated. (2) The efficacy of the small molecules identified in this study has not been further validated. Thus, in future research, we will focus on investigating the precise molecular mechanisms by which IGF1R influences MG using a mouse model and will validate the efficacy of the identified small molecules.

## 5 Conclusions

This study identified an association between low UA levels and MG through a meta‐analysis and subsequently used MR to identify the key gene, IGF1R. By leveraging datasets from GEO and TCGA, we validated the relationship between IGF1R and MG, showing that low IGF1R expression is associated with the onset, crisis, and poor prognosis of MG. Additionally, we evaluated the relationship between IGF1R expression and UA levels in MG patients using single‐center clinical data. Finally, virtual drug screening was employed to identify small molecules targeting IGF1R as potential therapeutic candidates for MG. Through this research, we aimed to elucidate the molecular mechanisms underlying the protective role of IGF1R in MG and identify potential drug candidates for MG treatment.

## Author Contributions

Y.S. and D.H. designed this study and wrote the draft of this paper. Y.S., L.P., H.W., and M.Z. contributed to the analysis and interpretation of data and assisted in the preparation of this paper. Q.H., W.W., S.L., Z.S., Y.F., H.C., Y.Q., D.T., and Y.S. contributed to data collection and interpretation of this paper. All of the authors approved the final version of this paper and agree to be accountable for all aspects of this paper.

## Disclosure

Consent to Participate: Informed consent was obtained from the subject involved in the study.

## Ethics Statement

The research was approved by the ethics committee of the first affiliated hospital of Nanchang University (AG‐SG‐03‐2.1‐IIT). This study acts in accordance with the ethical standards set forth in the 1964 Declaration of Helsinki and its subsequent amendments.

## Consent

Informed consent was obtained from individual participants included in the study.

## Conflicts of Interest

The authors declare no conflicts of interest.

## Data Availability

The data that support the findings of this study are available from the corresponding author upon reasonable request.
